# Resolvin E1 Regulates Th17 Function and T Cell Activation

**DOI:** 10.3389/fimmu.2021.637983

**Published:** 2021-03-17

**Authors:** Fatma Oner, Carla Alvarez, Wael Yaghmoor, Danielle Stephens, Hatice Hasturk, Erhan Firatli, Alpdogan Kantarci

**Affiliations:** ^1^ The Forsyth Institute, Cambridge, MA, United States; ^2^ Department of Periodontology, School of Dentistry, Istanbul University, Istanbul, Turkey; ^3^ Faculty of Dentistry, Universidad de Chile, Santiago, Chile; ^4^ Faculty of Dentistry, Umm Al-Qura University, Makkah, Saudi Arabia; ^5^ School of Dental Medicine, Harvard University, Boston, MA, United States

**Keywords:** RvE1, T helper (T) 17 cells H, resolution of inflammation, resolvin, T cell - DC interactions

## Abstract

Resolvin E1 (RvE1) is a specialized pro-resolving lipid mediator derived from eicosapentaenoic acid and plays a critical role in resolving inflammation and tissue homeostasis. T_h_17 cells are a distinct group of T helper (T_h_) cells with tissue-destructive functions in autoimmune and chronic inflammatory diseases *via* the secretion of IL-17. Dendritic cell (DC)-mediated antigen presentation regulates the T_h_17-induced progression of inflammation and tissue destruction. In this study, we hypothesized that the RvE1 would restore homeostatic balance and inflammation by targeting the T_h_17 function. We designed three experiments to investigate the impact of RvE1 on different phases of T_h_17 response and the potential role of DCs: First CD4^+^ T cells were induced by IL-6/TGF_β_ to measure the effect of RvE1 on T_h_17 differentiation in an inflammatory milieu. Second, we measured the impact of RvE1 on DC-stimulated T_h_17 differentiation in a co-culture model. Third, we measured the effect of RvE1 on DC maturation. RvE1 blocked the CD25, CCR6 and IL-17 expression; IL-17, IL-21, IL-10, and IL-2 production, suggesting inhibition of T cell activation, T_h_17 stimulation and chemoattraction. RvE1 also suppressed the activation of DCs by limiting their pro-inflammatory cytokine production. Our findings collectively demonstrated that the RvE1 targeted the T_h_17 activation and the DC function as a potential mechanism for inflammatory resolution and acquired immune response.

## Introduction

The resolution is critical for homeostatic balance to counteract the chronicity of inflammation (1, 2). Specialized pro-resolving lipid mediators (SPMs) regulate the cellular and molecular mechanisms to terminate the inflammatory process and restore pre-inflammatory conditions of health. This process, known as the resolution of inflammation, is a highly programmed and active stage mediated by lipoxins, maresins, protectins, and resolvins (3, 4). Resolvins are biosynthesized from omega-3 polyunsaturated fatty acids where E-series are derived from eicosapentaenoic acid, and D-series are derived from docosahexaenoic acid (5, 6). Studies in humans have demonstrated an association between defective levels of resolvins and inflammatory diseases (7, 8) and therapeutic effects of omega-3 polyunsaturated fatty acids in chronic inflammatory diseases (9). In preclinical studies, RvE1 prevented bone loss and regenerated tissue architecture (10, 11) by blocking osteoclast differentiation (12) through the RANKL/OPG pathway (13). The impact of RvE1 and other resolvins on neutrophils and macrophages suggests a de-regulatory function on the acute inflammation and innate immunity (14). The therapeutic and regenerative effects of RvE1 in animal models also imply that the impact of the SPMs is not limited to the prevention of acute inflammation from becoming chronic. The chronicity of the inflammatory response is highly regulated by the antigen-presenting cells such as dendritic cells (DCs) and subsequent activation of the T helper lymphocytes (T_h_) (15). In line with this approach, promoting effects of Lipoxin A4 (LXA4) and leukotriene B4 (LTB4) on T follicular helper cells show the role of SPMs on antigen-specific immune response (16) and functionally active receptor expression of LTB4 (BLT1) on effector T cells may link early immune activation and acquired immune response (17). Thus, it is plausible that the therapeutic effects of the SPMs would involve a direct impact on the DC-T_h_ axis.

In chronic inflammatory diseases, T lymphocytes control and regulate the host response by taking advantage of their memory properties and high cytokine-producing capacities (18). T lymphocytes are categorized into a variety of T helper (Th) subgroups, including T_h_1, T_h_2, T_h_17, and regulatory T cells (Treg), showing anti-inflammatory or pro-inflammatory properties (19) among these subgroups. T_h_17 cells have protective roles in controlling mucosal infections while presenting destructive functions in autoimmune and chronic inflammatory diseases (20, 21). T_h_17 cells further acquire phenotypic instability and transforming capacity from one cell to another, explaining their dual roles in inflammatory processes (22). T_h_17 cells produce a wide range of cytokine profiles, including IL-17, IL-21, IL-23, IL-22, IL-26, IL-6, IL-1βTGF_β,_ TNF_α_ and GM-CSF of which IL-17 is pivotal for their pro-inflammatory activation (23–25). As in the case of the other T cells, T_h_17 cells require antigen presentation for activation. Dendritic cells (DC) located at mucosal surfaces or in circulation are critical for T_h_17 cell function (26, 27). It is unknown how DC-induced T_h_17 cell activity is impacted during the resolution of the inflammatory process, which is an essential step for the homeostatic restoration.

In this study, we hypothesized that as a pro-resolution mediator, RvE1 will suppress T_h_17 differentiation, and this will be mediated through the DC-activity. To test this hypothesis, we analyzed the impact of RvE1 on several stages of T_h_17 activation, including T_h_17 polarization and DC-induced naïve T cell activation.

## Materials and Methods

### Reagents

CD11c Microbeads UltraPure kit, CD4+ T cell isolation kit, T cell medium (TexMACs), TGF_β_ recombinant protein and anti-chemR23 (APC) were purchased from MACS Miltenyi Biotec. Purified anti-mouse CD3 and CD28, recombinant mouse IL-6 (carrier-free), monoclonal anti-mouse antibodies; CD4 (APC), CD25 (FITC), *CCR6 (PE-Cy7)*, FoxP3 (Pacific Blue), IL-17 (R-PE), I-A^k^ MHC class II (PE), CD40 (PE/Cy5), CD86 (FITC) and CD80 (APC) were purchased from Biolegend. RORγt antibody (PerCP-Cy5.5) was purchased from R&D system, ionomycin and PMA were purchased from Sigma Aldrich. Dilutions of antibodies were as follows: RORγt was at 1/100; CD4, IL-17, CD40, and MHC II were at 1.25/100; CD25, CD86, and FoXP3 were at 2/100; CCR6 was at 5/100, and chemR23 was at 10/100. Monensin (1×) was purchased from Thermofisher Scientific. Pam_3_CSK_4_ was purchased from InvivoGen. RvE1 was purchased from Cayman Chemicals; RvE1 was stored in -80°C and immediately before use diluted in ethanol for the final concentration. IL-2 LUMINEX kit was purchased from Millipore Sigma. AYOXXA multiplex analysis kit was purchased from AYOXXA (Cologne, Germany).

### Animals

Male and female FVB, BALB/CBYJ type mice, were purchased from Jackson Laboratories and Charles River Laboratories. All animals were housed under standard pathogen-free conditions and kept feeding at the Forsyth Institute Animal Facility, Cambridge, MA. Six to ten-week-old mice were used in the experiments. The protocols were approved by the Forsyth Institute’s Institutional Animal Care and Use Committee (IACUC).

### Cell Purification and Sorting

Wild type mice without any genetic differences were sacrificed, and their spleen was excised. Spleen specimens were ground in sterile PBS with ACK lysis buffer (NH_4_Cl, Na_2_EDTA, KHCO_3_, ph 7.4) to eliminate red blood cells and filtered using 70 µm and 40 µm nylon mesh filters to achieve purity of splenocytes. Splenocytes were then passed through immune-magnetic depletion for specific cell type isolation. CD11c+ dendritic cells were isolated by CD11c MicroBeads UltraPure kit by positive selection through a midiMACS separator. CD4+ T cells were isolated by CD4+ T cell isolation kit (including monoclonal antibodies against CD8a, CD11b, CD11c, CD19, CD45R (B220), CD49b (DX5), CD105, Anti-MHC Class II, Ter-119, and TCRγ/δ) by negative selection through midiMACS separator. CD4^+^ T cells and CD11c^+^ dendritic cells had purity over 95%, confirmed by flow cytometry.

### T_h_17 Polarization

To identify the impact of RvE1 on T cell activation, we generated three different groups; 1) Unstimulated T cells, 2) T cell activation group, 3) RvE1 treatment group. 10^5^ CD4+ T cells were incubated in 200 µl final T cell medium (TeXMACS) with 10% FBS and 1% Penicillin-Streptomycin in triplicate for 5 days. For T cell activation, U-bottom 96-well plates were covered with 30 µl of anti-CD3(10 µg/ml) in 200 µl total volume with sterile PBS. After 4 hours incubation period at 37°C, plates were washed with 200 µl sterile PBS twice, and 1.5 µg/ml anti-CD28-alone was added to the medium for the next days. For RvE1 treatment; additionally, 10 nM RvE1 was added to the assigned groups on day 0 and 3. To evaluate the effect of RvE1 on T_h_17 polarization, we tested the following experimental groups: 1) Unstimulated T cells, 2) T_h_17 cell polarization group, 3) 10 nM RvE1 treatment group. Highly purified naïve CD4+ T cells were polarized into T_h_17 cells as described (28). Briefly, 10^5^ CD4+ T cells were incubated in 200 µl final T cell medium (TeXMACS) with 10% FBS and 1% Penicillin-Streptomycin in triplicate for each condition. TeXMACS is a serum-free cell culture medium without animal-derived components and contains pre-selected human serum albumin, stable glutamine, and phenol red. It provides enhanced T cell viability, consistency, and growth (29). In polarization and RvE1 treatment groups, U-bottom 96-well plates were covered with 30 µl of anti-CD3(10 µg/ml) in 200 µl total volume with sterile PBS. After 4 hours incubation period at 37°C, plates were washed with 200 µl sterile PBS twice. For the T_h_17 polarization group, a mixture of 20 ng/ml IL-6, 5 ng/ml TGFβ and 1.5 µg/ml anti-CD28 were prepared, and 100 µl of this mix were added to each well. For the RvE1 treatment group, in addition to the T_h_17 polarization mix, 10 nM RvE1 was added on day 0 and 3. All cells were incubated at 37°C for five days. Cells for FACS analysis and supernatants for Multiplex analysis were collected on day 5.

### T_h_17 Stimulation by DC

To determine the ability of DCs to stimulate naïve CD4+ T cells into T_h_17 cells and evaluate the impact of RvE1, we co-cultured naive CD4+ T cells (50,000/200 µl) with CD11c+ DC (1000/200 µl) in U-bottom 96-well plates in 200 μl RPMI containing 10% FBS and 1% Penicillin-Streptomycin. Recent publications show that toll-like receptor (TLR) signaling regulated by DCs was sufficient for T helper cell stimulation and Th17 differentiation (30, 31); Pam_3_CSK_4_ is a TLR activator necessary for Th17 polarization (32, 33). Thus, Pam_3_CSK_4_ (100 ng) was used to stimulate cells. Six experimental groups were tested: 1) Unstimulated CD4+ T cells 2) CD4+ T cells+ Pam_3_CSK_4_ 3) DCs+CD4+ T cells 4) DCs+CD4+ T cells+ RvE1 5) DCs+CD4+ T cells+ Pam_3_CSK_4_ 6) DCs+ CD4+ T cells+ Pam_3_CSK_4_+ RvE1. All cells were incubated at 37°C for 5 days. First, cells were treated with 10 nM RvE1 for 24 hours and then stimulated with 100 ng Pam_3_CSK_4_. Another dose of RvE1 was applied to the cells on day 3. Cells and supernatants were collected for FACS analysis and Multiplex analysis on day 5.

### DC Maturation

CD11c+ DCs were stimulated with Pam_3_CSK_4_ in the presence or absence of RvE1 to evaluate its impact on the activation of DC. 2x10^5^ cells were incubated in 48-well plates in 500 μl RPMI containing 10% FBS and 1% Penicillin-Streptomycin at 37°C for three days. First cells were treated with 10 nM RvE1 for 24 hours, and then 100 ng Pam_3_CSK_4_ was added. An additional dose of RvE1 was applied on day 2. Cells-alone or treated only with RvE1 were used as control. All cells were collected for FACS analysis on day 3.

### Expression of Surface Receptors and Antigens on Th Cells and DCs

Cell viabilities were checked by trypan blue before analyzing surface receptor expressions at the end of the experiments. CD4, CD25, CCR6, chemR23, FoxP3, RORγt and IL-17 expressions on T cells were measured on day 5. T cells in triplicate were pooled in 24-well culture plates in 1 ml final volume for each condition. PMA (phorbol myristate acetate) (50 ng/ml), ionomycin (1µM) and monensin (1×) were added to each well for 6 hours at 37°C. CD4, CD25, and CCR6 antibodies were added to the cells for 30 minutes on ice. After washing, cells were fixed with 4% paraformaldehyde for 10 minutes at room temperature. Then, 0.1% Triton for permeabilization and 0.5% BSA for blocking was applied for 10 minutes at room temperature and on ice, respectively. Antibody cocktail containing FoxP3, RORγt, and IL-17 were added for intracellular staining, and FACS analysis was performed. CD80, CD86, CD40, MHC II expressions on DC were measured on day 3; antibody cocktail was added to cells for 30 minutes on ice. Then, 4% paraformaldehyde, 0.5% BSA, and 0.1% Triton was applied as described above before performing FACS analysis. For all the experiments, the cell population was selected in the FSC/SSC plot according to their expected cell size and granularity. Singlets were gated to eliminate doublets and then proceeded to the individual analysis of the markers. All analyses were made by Attune NXT Flow Cytometer Software.

### Multiplex Analysis

To determine the cytokine release, IL-2, IL-17A, IL-17F, IL-21, CCL20, IL-6, and TGF_β_ were measured. IL-2 assay was performed according to the manufacturer’s instructions (Millipore) and assayed on Luminex (BioRad). The other cytokines were measured by AYOXXA multiplexing, as described by the manufacturer.

### Statistical Analyses

ANOVA was used for statistical analyses. Tukey’s posthoc analysis was used for multiple comparisons. Differences were considered statistically significant when p was less than 0.05.

## Results

**Figure 1:**
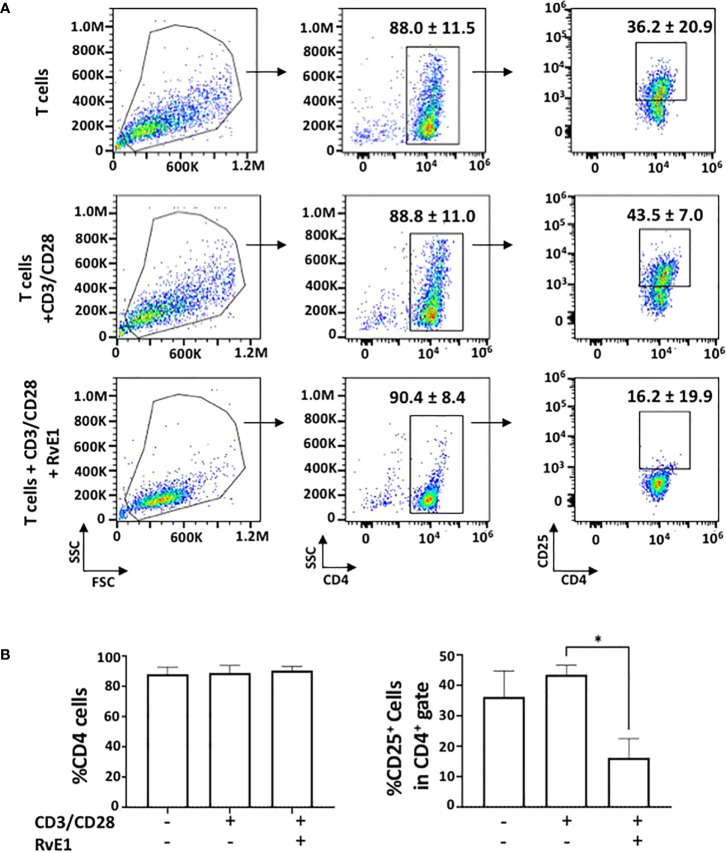
RvE1 suppresses T cell activation by decreasing CD25^+^ cells. **(A)** There is an example of gating strategies **(A)** shows representative flow cytometric data and **(B)** shows proportion of CD4^+^ cells and CD25^+^ cells in CD4^+^ gate in unstimulated T cells, activated T cells and RvE1 treatment groups Data showed are ± sem for six independent experiments. *p < 0.05.

To test the impact of RvE1 on T cell activation, naive CD4+ T cells obtained from the spleen were activated with anti-CD3 and soluble anti-CD28 in the presence or absence of RvE1. PMA and ionomycin were added for 6 hours on day five, and FACS analysis was performed. RvE1 did not change CD4+ T cell expressions; the frequency of CD4+ T cells was high without any noticeable difference between groups. Meanwhile, CD25+ T cells in response to RvE1 were decreased significantly (**Figure 1**). There was no statistical change in the viability of cells due to stimulation or RvE1 treatment.

### RvE1 Suppresses T_h_17 Proliferation Under Polarizing Conditions

**Figure 2:**
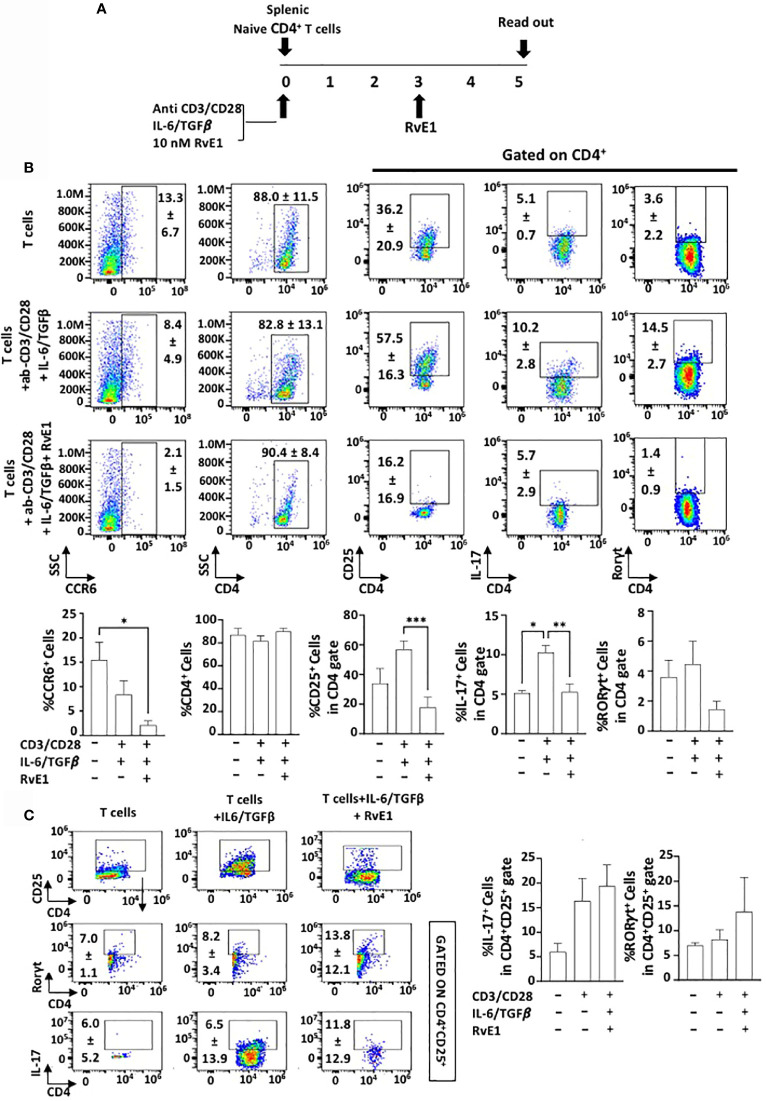
RvE1 prevents Th17 differentiation by inhibiting IL-17 expression and T cell activation by decreasing CD25 expression. RvE1 regulates the migration of Th17 cells by decreasing CCR6 expression. **(A)** Experimental design protocol. Naïve CD4^+^ T cells were stimulated to Th17 cells under Th17 polarizing conditions. T cells were cultured alone for control groups. For the Th17 stimulation group, ab-CD3/CD28 and IL-6/TGF*β* was applied, and for the RvE1 treatment group, besides these polarizing conditions 10 nM RvE1 was used baseline and on day 3. All cells were incubated for five days at 37°C. Cells were induced with PMA/ionomycin and monensin for 6 hours on day five and analyzed by flow cytometry. **(B)** Representative flow cytometric data (above) and proportion (below) of CCR6^+^ cells, CD4^+^ cells and CD25^+^, IL-17^+^ and RORγt^+^ cells in CD4^+^ gate in unstimulated T cells, Th17-polarized cells and RvE1 treatment groups. **(C)** shows flow cytometric data (left) of CD4^+^CD25^+^ cells, IL-17^+^ and RORγt^+^ cells gated in CD4^+^CD25^+^ cells and proportion (right) of IL-17^+^ and RORγt^+^ cells gated in CD4^+^CD25^+^ in unstimulated T cells, Th17-polarized cells and RvE1 treatment groups. Results are ± sem for six independent experiments *p < 0.05, **p < 0.01, ***p < 0.001.

Splenic CD4^+^ T cells were polarized into T_h_17 cells under specific polarizing conditions (CD3/CD28 activation and IL6/TGFβ stimulation). RvE1 was applied baseline and on day 3. Cells were cultured for five days, and data was analyzed (**Figure 2A**). CD4^+^ T cell percentage was similar in all experimental groups (**Figure 2B**). To determine the impact of RvE1 on T_h_17 stimulation from naive CD4+ T cells, we analyzed RORγt and IL-17 expressions. Th17 polarizing increased the expression of RORγt and IL-17; RvE1 prevented the impact of polarization and significantly decreased IL-17^+^ cells. Simultaneously, there was a decrease in RORγt^+^ cells in response to RvE1, the difference was not statistically significant. We also analyzed the cc chemokine receptor 6 (CCR6) and its ligand CCL20 to measure the chemotactic migration of T_h_17 cells. The highest CCR6 expression was observed in unstimulated cells, and RvE1 decreased these levels significantly. RvE1 resulted in a significant decrease in CD25 expression by the T_h_ cells. CD25 expression was observed in unstimulated groups and reached a peak level after the polarization. RvE1 prevented the expression of these receptors. To analyze the impact of RvE1, we then gated CD4^+^CD25^+^ cells and analyzed IL-17 and RORγt expressions. RvE1 decreased the frequency of CD4^+^CD25^+^ cells, but in contrast with its effect gated on only CD4^+^ cells, there was an increasing trend on IL-17^+^ and RORγt^+^ cells in response to RvE1 with no statistical significance (**Figure 2C**).

**Figure 3:**
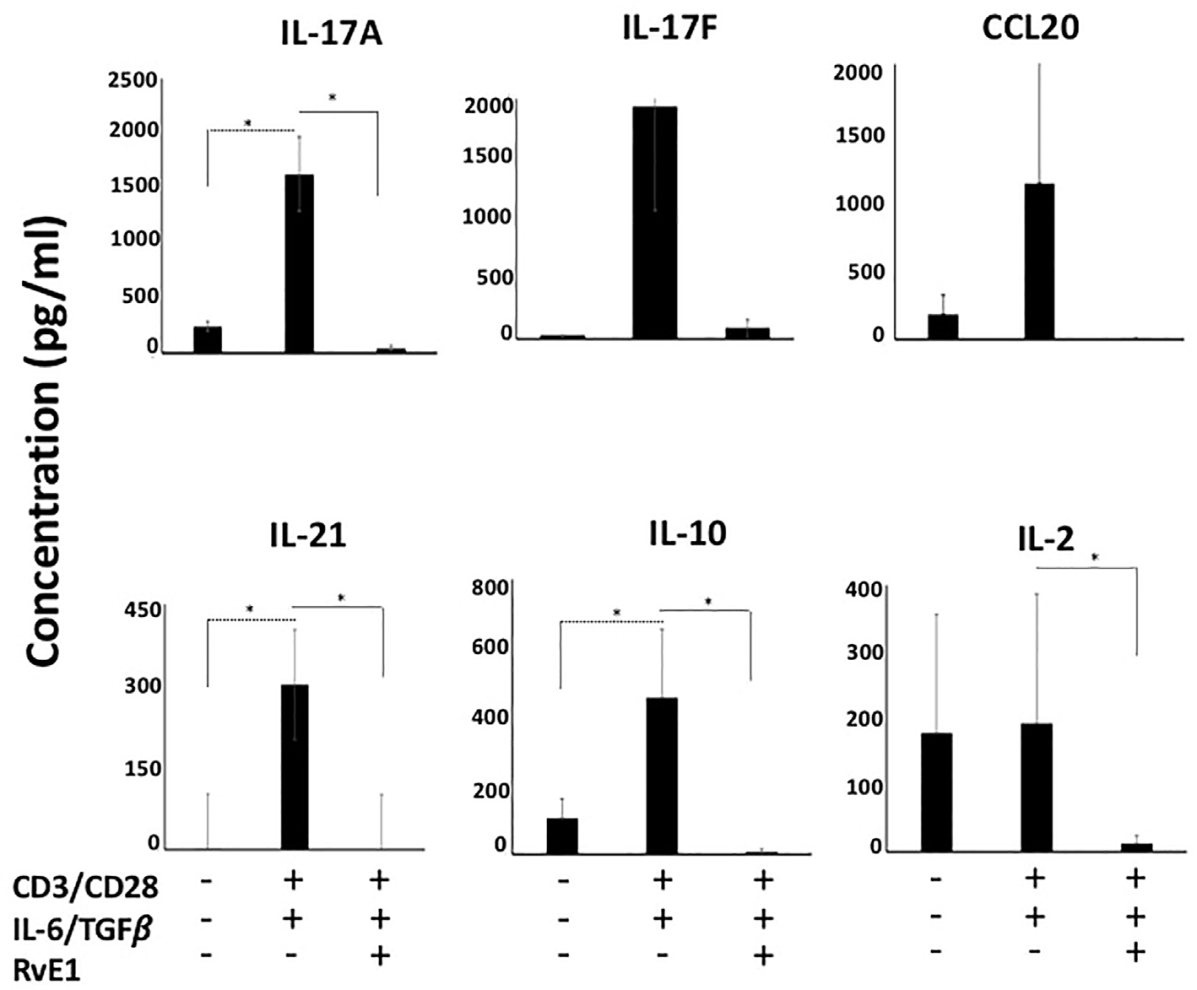
RvE1 suppresses cytokine secretion from Th17 cells. IL-17A, IL-17F, CCL20, IL-21, IL-10 and IL-2 secretions from unstimulated T cells, Th17-polarized cells and RvE1 treatment groups were analyzed. IL-17A, IL-17F, CCL20, IL-21 and IL-10 were analyzed by AYOXXA and IL-2 was analyzed by Luminex. Results are ± sem for six independent experiments, *p < 0.05.

We then measured IL-17A, IL-17F, IL-21, and IL-10 content in supernatants (**Figure 3**). The highest cytokine secretion was observed by CD4 polarized T_h_17 cells; levels of IL-17A, IL-17F, IL-21, and IL-10 were limited in unstimulated groups. There was a substantial increase in their secretion levels after polarization. RvE1 led to a significant decrease in IL-17A. IL-17F decline was also noteworthy, while the change was not statistically significant. IL-10 and IL-21 generation by the T_h_17 cells significantly decreased in response to RvE1. Consistent with the CCR6 expression, RvE1 reduced the CCL20 secretion to the levels of the unstimulated group. We also analyzed the levels of secreted IL-2, which, in line with its receptor (CD25) activation, showed a significant decrease after RvE1 treatment.

### Effect of RvE1 on DC-Stimulated T_h_17 Proliferation

**Figure 4:**
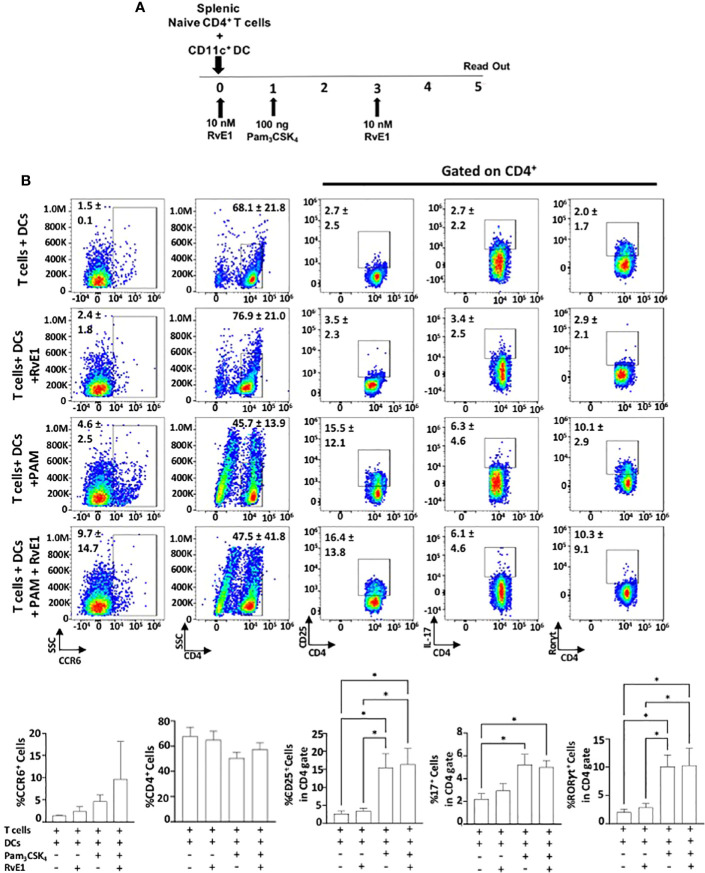
**(A)** Experimental design protocol. CD4^+^ T cells were co-cultured with dendritic cells (1000 DC/50000 T cell) in the presence or absence of PAM_3_CSK_4_ (100 ng) and RvE1 (10 nM). RvE1 was applied baseline and on day 3; PAM_3_CSK_4_ was applied on day 1. All cells were incubated at 37°C for 5 days **(B)** Representative flow cytometric data (above) and proportion (below) of CCR6^+^ cells, CD4^+^ cells and CD25^+^, IL-17^+^, RORγt^+^ cells in CD4^+^ gate in T cells+ DCs, T cells+DCs+RvE1, T cells+ DCs+ PAM_3_CSK_4_ and T cells+ DCs+ PAM_3_CSK_4_+ RvE1. Results are ± sem for four independent experiments, *p < 0.05.

**Figure 5:**
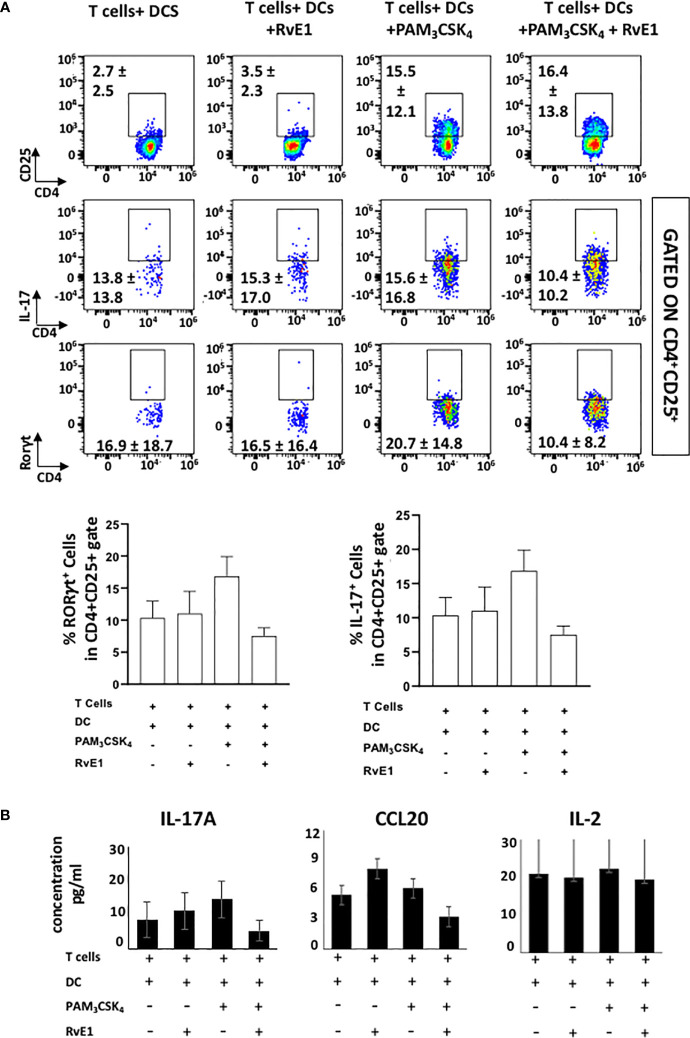
**(A)** shows flow cytometric data (above) of CD4^+^CD25^+^ cells, IL-17^+^ and RORγt^+^ cells gated in CD4^+^CD25^+^ cells and proportion (below) of IL-17^+^ and RORγt^+^ cells gated in CD4^+^CD25^+^ cells T cells+ DCs, T cells+DCs+RvE1, T cells+ DCs+ PAM_3_CSK_4_ and T cells+ DCs+ PAM_3_CSK_4_+ RvE1. **(B)** shows IL-17A, CCL20 and IL-2 secretions by Th17 cells. CCL20 and IL-17A were analyzed by AYOXXA and IL-2 was analyzed by LUMINEX. Results are ± sem for four independent experiments.

We analyzed the impact of RvE1 when naive CD4^+^ T cells were stimulated by DCs (**Figures 4** and **5**). As the optimal IL-17 production by the T_h_17 cells was reported in the presence of Pam_3_CSK_4_ in co-culture experiments (34), we incubated CD11c^+^ DCs with naïve CD4^+^ T cells (1000 DC/50000 T cells in 200 µl total medium) in the presence of Pam_3_CSK_4_ (100 ng) with or without RvE1 (10 nM) for five days. RvE1 was applied to the cells for 24 hours, and then Pam_3_CSK_4_ was added (**Figure 4A**). Before performing the FACS analysis, PMA, ionomycin, and monensin were added for 6 hours. The percentage of CD4^+^ T cells was similar in each group (**Figure 4B**). To evaluate the impact of RvE1 on T_h_17 stimulation, we analyzed RORγt and IL-17 expressions. Pam_3_CSK_4_ stimulation significantly increased RORγt^+^ cells; RvE1 application did not cause any further change in these levels, same as its effect on IL-17^+^ cells. We then analyzed IL-17A secretion from Th17 cells. Pam_3_CSK_4_ stimulation increased IL-17A levels, which were decreased in response to RvE1, while the changes were not statistically significant (**Figure 5B**). Pam_3_CSK_4_ stimulation and RvE1 treatment increased the CCR6 expression (**Figure 4B**) in contrast with its ligand-CCL20 that decreased after RvE1 treatment (**Figure 5B**); the changes due to RvE1 was not statistically significant (**Figures 4B** and **5C**). We also stimulated T cells alone with Pam_3_CSK_4_ and measured RORγt^+^ and IL-17^+^ cells. Pam_3_CSK_4_ stimulated RORγt^+^, IL-17^+^ cells expressed by T cells independently from DCs (data not shown) similarly suggesting a toll-like receptor signaling on T cells that may promote T helper cell differentiation independent from antigen-presenting cells (33, 34). In a similar pattern, Pam_3_CSK_4_ stimulation significantly increased CD25 expression but differing from CD4-polarized Th17 cells applying RvE1 did not affect these levels (**Figure 4B**). same as its effects on IL-2 secretion. IL-2 secretions by T cells co-cultured with DCs were at similar levels independent from Pam_3_CSK_4_ stimulation or RvE1 treatment (**Figure 4C**). We then gated CD4^+^CD25^+^ T cells; similar to CD25 expressions, CD4+CD25+ cells slightly increased in response to RvE1. A decreasing trend was observed on IL-17 and RORγt expressions by CD4^+^CD25^+^ T cells (**Figure 5A**).

### ChemR23/ERV1 Receptor Is Expressed on DCs and T_h_17 Cells

**Figure 6:**
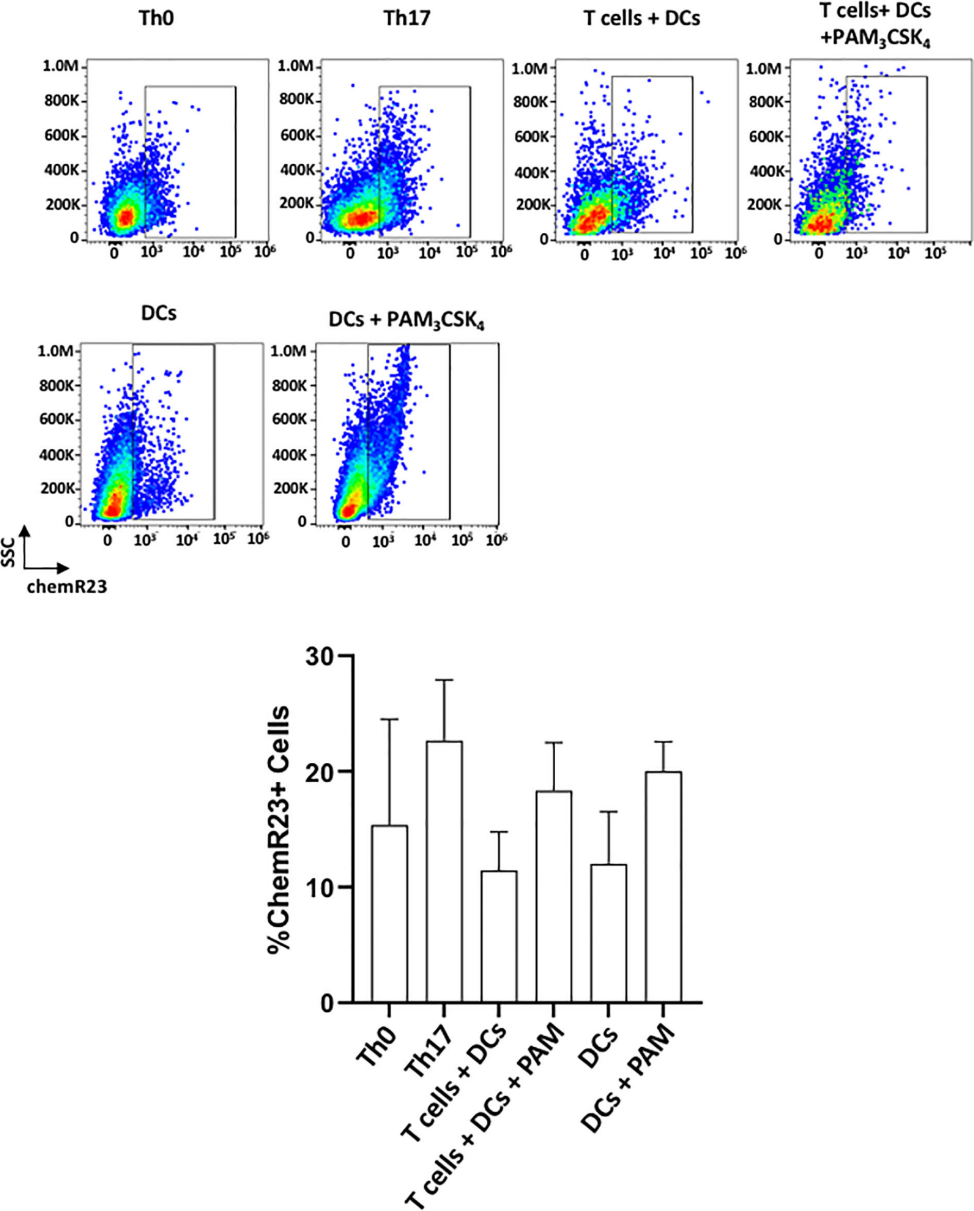
chemR23/ERV1 is expressed on T cells^+^ and DCs. Representative flow cytometric data (above) and proportion (below) of cells showing chemR23 expression on Th0 cells (CD4^+^ T cells + ab-CD3/CD28), Th17 cells (CD4^+^ T cells+ ab-CD3/CD28+ TGFβ/IL-6), T cells-DCs coculture, T cells-DCs co-culture with PAM_3_CSK_4_ stimulation, DCs and DCs with PAM_3_CSK_4_ stimulation. Results shown are ± sem for three independent experiments.

**Figure 6** shows the expression of ChemR23/ERV1 (receptor for the RvE1) on DCs and T cells. ChemR23 expression was previously reported on DCs and lymphocytes (35). In line with previous studies, both T cells and DCs expressed substantial levels of receptors for SPMs in unstimulated/stimulated conditions (36, 37). Th17-polarized cells expressed more receptors compared with Th0 (CD3/CD28 activated T cells). In a similar pattern, Pam_3_CSK_4_ stimulated the expression of chemR23 on both T cells and DCs. (**Figure 6**). The overall changes, however, were not statistically significant among groups.

### Effect of RvE1 on FoXP3 Levels

**Figure 7:**
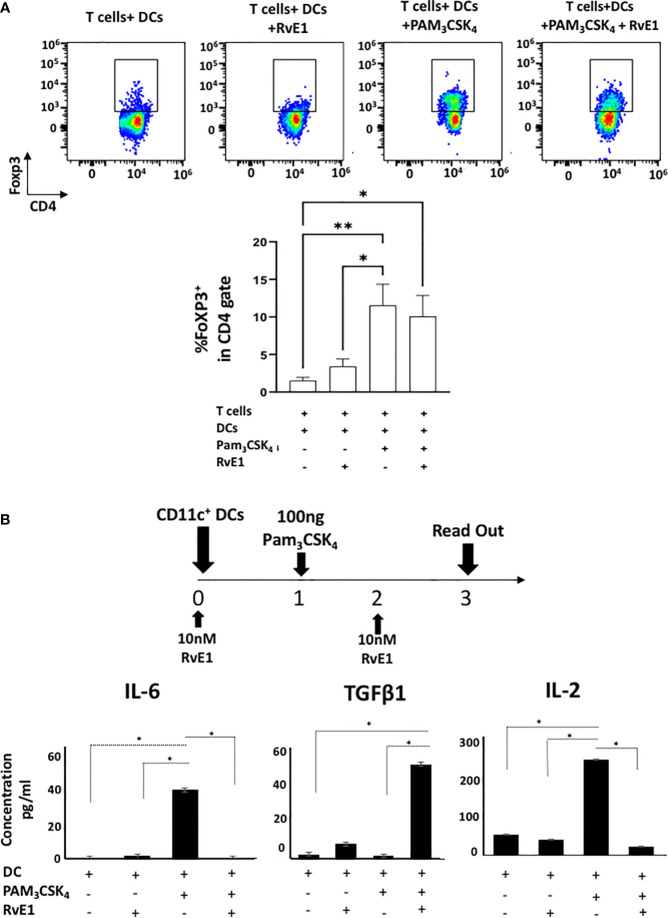
**(A)** shows representative flow cytometric data (above) and proportion (below) of FoXP3^+^ cells in CD4^+^ gate in T cells+ DCs, T cells+DCs+RvE1, T cells+ DCs+ PAM_3_CSK_4_ and T cells+ DCs+ PAM_3_CSK_4_+ RvE1. Naive CD4^+^ T cells were cocultured with dendritic cells and stimulated by PAM_3_CSK_4,_ RvE1 treatment was applied baseline and on day 3 to the assigned groups. Cells were incubated at 37°C for five days, and on day 5. PMA/ionomycin and monensin were applied to the cells for 6 hours before performing FACS analysis. **(B)** CD11c+ dendritic cells (2x10^5 cells/500 𝓂l) were treated with 10 nM RvE1 for 24 hours and then induced with 100 ng PAM_3_CSK_4_ for 48 hours. One more dose of RvE1 was applied on day 2. **(B)** shows secreted cytokine levels by dendritic cells; Supernatants were collected on day 3; IL-6 and TGF_β_ were analyzed by AYOXXA and IL-2 by LUMINEX. Results shown are means ± sem for at least four independent experiments *p < 0.05, **p < 0.01.

FoXP3 is the transcription factor necessary for differentiation of regulatory T cells (Treg), which are crucial to prevent autoimmune disorders and the extent of inflammation (38) with suppressive functions on Th17 cells (39). **Figure 7** demonstrates FoXP3 expression on DC-stimulated T helper cells. There was a limited FoXP3 expression on T cells co-cultured with DCs; this was significantly increased with Pam_3_CSK_4_ stimulation where RvE1 did not further change the percentage of FoXP3^+^ cells (**Figure 7A**). We also measured FoXP3 expression in CD4-polarized Th17 cells; however, very low expressions were detected as expected (data not shown).

### RvE1 Suppresses the Activation of DCs

Our results showed the effect of RvE1 on T_h_17 polarization either directly by specific T_h_17 proliferative reagents or by DC stimulation. Thus, we measured the effect of RvE1 on the maturation and activation of DCs. DCs were stimulated with Pam_3_CSK_4_ (100 ng) with or without RvE1 (10 nM) for three days and analyzed (**Figure 7B**). CD80/86, CD40, and MHC II expressions were analyzed by flow cytometry to measure the DC maturation and the effect of RvE1. Although expression of CD40, CD80, and MHC II increased after PAM_3_CSK_4_ stimulation, reduction by the RvE1 was not significant (**Supplementary Figure 1**). We then examined IL-6, IL-2, and TGFβ content secreted by DCs; secretion of these cytokines was limited in DCs; after stimulation with Pam_3_CSK_4,_ there was a substantial and significant increase in IL-6 and IL-2 levels, which RvE1 significantly reduced. TGFβ levels reached peak levels after RvE1 treatment in T cell-DCs groups stimulated with Pam_3_CSK_4_ (**Figure 7B**).

## Discussion

Since the discovery of RvE1 in exudates from murine dorsal pouches treated with aspirin and EPA (40), resolvins and other SPMs have been extensively used as mediators of resolution in chronic inflammatory diseases. Many of these studies focused on the cellular and molecular mechanisms of innate immunity. We tested the hypothesis that the RvE1 will restore homeostatic balance and inflammation by targeting the T_h_17 activation, a critical step in the chronicity of inflammation. We used the splenocyte-derived CD4^+^ T cells and CD11c^+^ DCs (41–45) and minimized inter-individual variations. We applied the RvE1 at baseline and day 3 since supplementary doses were necessary to show its impact. Administering the other day would be another way to test the effect (37), but as Th17 polarization is achieved either on day 3 or 5 (28); we decided to repeat the application on day 3 when we added a fresh medium to the cell culture and possibly providing a new model for future research. We used three different doses of RvE1 (1 nM, 10 nM, and 100 nM) and analyzed Th17 polarization from naive CD4^+^ T cells. These experiments were used to optimize the RvE1 dose in this study. The most efficient results were achieved by 10 nM RvE1; the effects of 1 and 100 nM doses were similar to 10 nM. None of the doses had any significant impact on the viability of cells. Therefore, we chose to use the 10 nM RvE1 treatment for the rest of the assays, including the DC-mediated Th17 proliferation and DC maturation. We only checked the viability of cells under the microscope and during FACS analysis. This is a limitation of our study; however, based on the flow dot plots in the figures, we can interpret that the viability of our cells was high.

Receptor-mediated production of cytokines is critical for T_h_17 function. RORγt receptor expression is necessary for effector T_h_17 cells and an efficient cytokine production such as IL-17 (23–25, 44–48). In addition to the IL-17, IL-21 is generated by T cells in the inflammatory milieu and provides an alternative pathway for pathogenic T_h_17 differentiation (49). In our study, RvE1 prevented IL-17 expression and IL-17A secretion by T_h_17 cells, consistent with the findings of RvD1, RvD2 and maresin (37), suggesting that the SPMs may use common intracellular and post-receptor signaling pathways regulating the T_h_17 function. However, these data contrasted with the findings that another SPM, LTB4, increases IL-17 secretion and RORγt expression suggesting a promoter effect on Th17 cells (50). Our data also showed a substantial increase in IL-21 levels when T_h_17 cells were polarized, where RvE1 completely prevented this increase and restored the IL-21 secretion. Meanwhile, IL-10, another critical anti-inflammatory cytokine (51), was decreased in response to RvE1. Currently, there is no consensus on the impact of resolvins on IL-10 secretion. While some studies suggested that resolvins increased IL-10 secretion (13, 37, 52), others reported an opposite effect (53). This could be due to a complex interplay between the pro-inflammatory and anti-inflammatory cytokine production by the T_h_17 cells; It is known that IL-1β increases IL-17 production while inhibiting IL-10 secretion (54). In the absence of an inflammatory milieu, however, the resolution phase-associated cytokines of the inflammation may be similarly reduced as the pro-inflammatory cytokines as a function of the non-pathogenic T_h_17 cell population, which secrete IL-10 cytokines (55, 56).

The effect of RvE1 on T_h_17 cells is not only limited to their proliferation capacity but also inhibition of their attractivity with decreased CCR6 receptor expression and CCL20 cytokine secretion. CCR6^+^ cell populations together with CXCR3^+^ cells produce both IL-17 and IFN-γ. Therefore, CCR6^+^ cells are also T_h_17/T_h_1 cells (57). Meanwhile Treg cells express CCR6 receptor but not CCL20 (58, 59). The highest CCR6 expression with almost no CCL20 secretion observed in unstimulated T cells may suggest that different T cell groups can express CCR6 but not CCL20. Our data suggested that RvE1 restore the chemoattraction of T_h_17 cells by targeting both CCR6 and CCL20 levels under T_h_17 polarizing conditions. In a recent *in vivo* study, RvE1 was shown to decrease the number of DCs and T_h_17 cells in the inflamed area without impacting the CCL20 levels (60), suggesting additional compensatory mechanisms regulating the CCR6 activation in tissues.

DC-T cell interaction is a highly organized process starting with the engagement of Toll-like receptor (TLR) and peptide-loaded major histocompatibility complex (MHC), following with the activation of co-stimulatory molecules CD40, CD80/CD86, and ending up with cytokine secretion (61). In DC-mediated T_h_17 polarization, the inhibitory impact of the RvE1 on RORγt, IL-17, and CD25 expression and cytokine secretion were not as effective as it was under T_h_17-polarizing conditions suggesting that DCs decrease the responsiveness of T cells against RvE1. The role of DCs in this system is not clear; our data revealed that Pam_3_CSK_4_ stimulation increased the IL-6 and IL-2 secretion by the DC, and RvE1 reversed this effect while increasing the TGF_β_ levels. A similar mechanism for the inhibition of T cell activation through blocking monocyte activation in health but not in chronic inflammatory conditions has been linked to Annexin A1, which acts as endogenous anti-inflammatory mediator (62). In parallel with our findings, other researchers have demonstrated that in response to RvE1, DC maturation was suppressed, IL-12 and TNFα generation was decreased and DCs generated from precursor molecules in the presence of RvE1 secreted less IL-17 from pre-activated T cells (52). In an *in vivo* sepsis study, RvD1 decreased IL-6, TNF_α_, IL-1_β_ and IFN_γ_ levels significantly (53). Overall, the DC-mediated T cell response to the SPMs seems to be pathology-specific and may differentially involve the activity of other cell types including the DCs in health and disease.

IL-2 production by CD4^+^ T helper cells is increased following antigen activation; CD25 expression increased during T cell activation increases the IL-2 receptor’s affinity for its ligands (63–65). Our work demonstrated that the stimulation of naïve CD4^+^ T cells with either Pam_3_CSK_4_ in co-cultures or Th17 polarizing reagents resulted in the most profound increase in CD25 expression; RvE1 significantly decreased this under T_h_17 polarizing conditions. This finding was in parallel with a significantly reduced IL-2 cytokine secretion by T cells under the same conditions by the RvE1, which may suggest that RvE1 suppressed IL-2 secretion leading to a decrease of CD25 expression (66). CD4^+^CD25^+^ T cells are known as “natural suppressor cells,” showing anergic, suppressing functions by inhibiting transcription of IL-2 *via* TCR, Exposing to antigen stimulation or polyclonal T cell receptor stimulation activates these cells (CD4^+^CD25^+^) to mediate their suppressive functions (67, 68), which may be the reason for increased CD4^+^CD25^+^ T cell populations under Th17 polarizing conditions and DC-mediated Th17 polarization. Our data showed that RvE1 does not have effect on CD4^+^CD25^+^ cells or on CD4^+^CD25^+^IL-17^+^ and CD4^+^CD25^+^RORγt^+^ cells. While further studies are required to elucidate the effector functions of these cells during the resolution phase of the inflammation, previous reports on CD25 expression by Th17 cells (CD25^+^CCR6^+^) showed anti-inflammatory properties *via* high CTLA1 expression and suppress the activity of CD8^+^ T cells and IFNγ production (69).

CD4^+^CD25^+^ T cells express FoxP3 to become regulatory T (Treg) cells and reach higher phenotypic stability; FoxP3 is a necessary transcription factor for Treg (CD4^+^CD25^+^FOXP3^+^) cells to maintain immunosuppressive and anti-inflammatory functions (70). Low levels of FoXP3 expression were detected under Th17 polarizing conditions as expected; however, FoXP3 was significantly increased in DC-mediated Th17 polarization showing that Treg cell populations also increased following Pam_3_CSK_4_ stimulation. Although it was not significant, there was a tendency towards decreasing the FoXP3 expression due to RvE1. It was previously reported that in the resolution of inflammation mediated by a non-SPM agonist-Annexin, there was no effect on Treg cell populations (CD4^+^FoXP3^+^) (62). While our data is not sufficient to conclusively predict the effect of RvE1 on Treg cells, as the IL-2 is critical for the generation, survival, and function of Treg cells (71), decreased CD25 and IL-2 levels by RvE1 may lead to lower FoXP3 expression. Collectively, these data suggested that RvE1 may inhibit Treg differentiation in addition to T_h_17 differentiation. SPMs may be expected to increase FoxP3 and Treg populations in a targeted polarization (37). Recently animal models demonstrated the regulatory function of Maresin 1 on Treg/Th17 balance through transcription factors (72, 73) suggesting a role for the inflammatory milieu in which T_h_17 and Treg cells work counterregulatory of each other’s function. Our data are in line with findings suggesting that IL-2-mediated mechanism by Treg cells may lead to an increase of both T_h_17 and Treg cells at the same time by preventing inhibition capacity of IL-2 over T_h_17 cells (74). In this scenario, a reduced IL-2 level may be preventing the IL-2-mediated suppression of RORγt expression and T_h_17 differentiation (75), suggesting that the IL-2 is an essential cytokine in RvE1-dependent regulation of T cell class switch.

**Figure 8:**
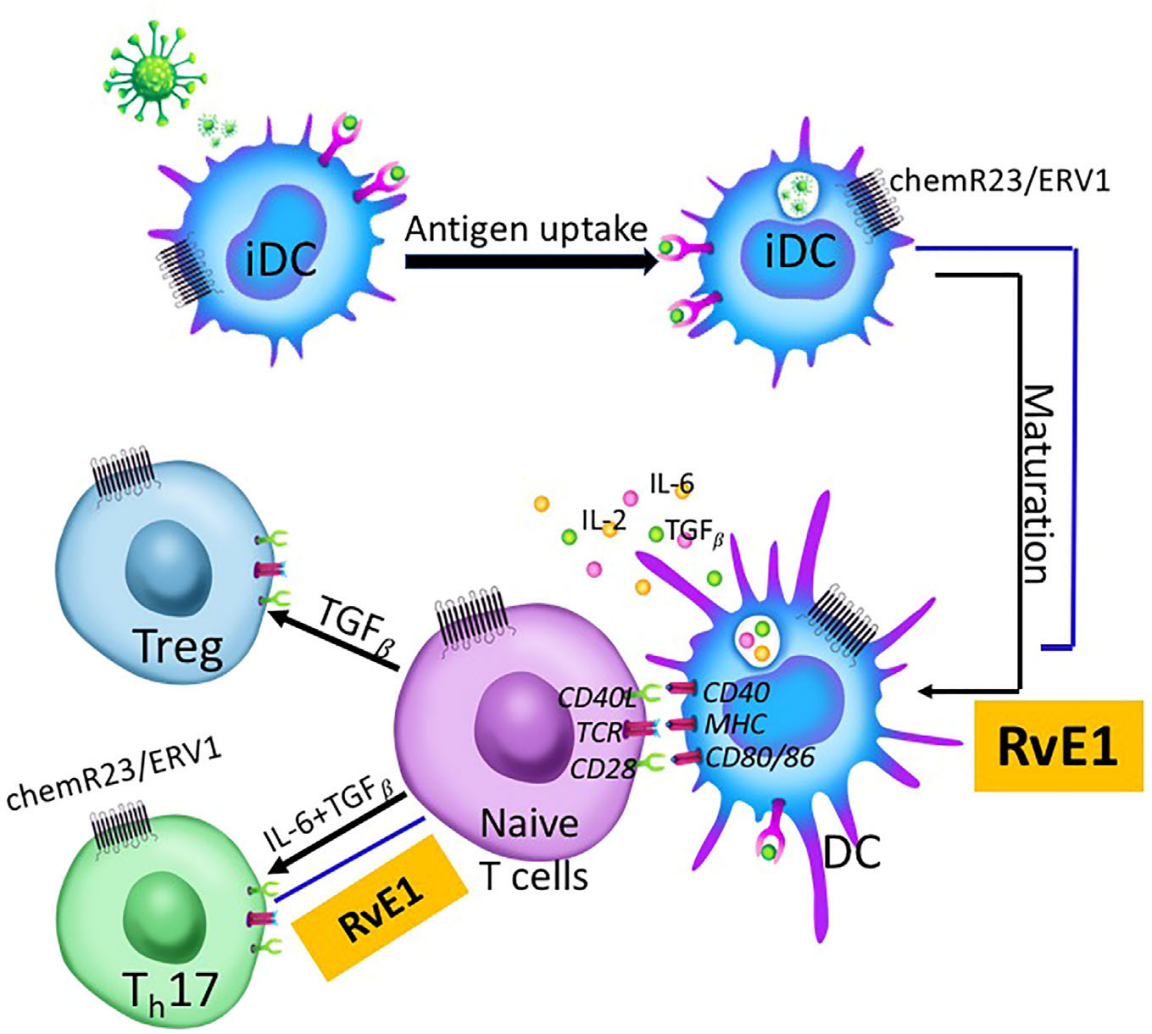
Immature dendritic cells (iDC) located at mucosal surfaces or in circulation are the antigen-presenting cells coming across with the antigen initially. After taking up the antigen, they undergo maturation and migrate to lymphoid tissues where naive T cells are present. DC-T cell cross-talk is a three-step process involving TCR-MHC binding and resulting in cytokine production. T cells differentiate into T helper subgroups for effector functions depending on the cytokines produced by DCs and T cells. IL-6 and TGF*β* induce T_h_17 proliferation while TGF*β-*alone induces Treg proliferation. RvE1 impacts this process at two different levels; 1) DC maturation, and 2) T_h_17 proliferation from naive T cells. RvE1 receptor chemR23/ERV1 is expressed both on DCs and T cells. RvE1 prevents DC maturation by regulating cytokine production from DCs, and T_h_17 differentiation from naive T cells, Th17 chemoattractiom and T cell activation.

Our data demonstrated that the RvE1 blocked the CD25, IL-17, and CCR6 expression; IL-17, IL-21, IL-10, and IL-2 production by T_h_17 cells. RvE1 prevented IL-6 and IL-2 production and stimulated TGF_β_ production by DC. The data present two critical outcomes: 1) SPMs regulate the chronicity of inflammation by reversing T_h_17 activation, and 2) Antigen-presenting DCs that are involved in T_h_17 activation could not be a therapeutic target in preventing the lymphocytic involvement of acquired immune responses during inflammation as they decrease the responsiveness of T cells (**Figure 8**).

## Conclusion

Immune response orchestrated by T cells is a highly complex process due to the engagement of different molecules. Our results showed that RvE1 inhibited T helper cell activation by decreasing CD25 expression while suppressing the T_h_17 proliferation. A parallel decrease in IL-6 and an increase in TGFβ secreted by DCs with a critical role of IL-2 suggested that the RvE1-mediated resolution of T_h_17 cell activity.

## Data Availability Statement

The original contributions presented in the study are included in the article/**Supplementary Material**. Further inquiries can be directed to the corresponding author.

## Ethics Statement

The animal study was reviewed and approved by Forsyth Institute`s Animal Care and Use Committee in accordance with Institutional Animal Care and Use Committee with Animal Assurance Number; A3051-01/D16-00029.

## Author Contributions

FO and AK conceptualized the study and wrote the original draft. FO performed the experiments. WY collected the animal samples. FO, CA and AK contributed to assembly data, FO, CA and DS performed data analysis. EF and HH provided administrative support. All authors contributed to the article and approved submitted version.

## Funding

This project was supported by the NIH R01AG062496 grant and doctoral award by the Scientific and Technological Research Council of Turkey (BIDEB 2214-105B141600394).

## Conflict of Interest

The authors declare that the research was conducted in the absence of any commercial or financial relationships that could be construed as a potential conflict of interest.

## Supplementary Material

Supplementary Figure 1 CD11c+ dendritic cells (2x10^5 cells/500 𝓂l) were treated with 10 nM RvE1 for 24 hours and then induced with 100 ng PAM_3_CSK_4_ for 48 hours. One more dose of RvE1 was applied on day 2. Cells were collected for analysis on day 3; antibody cocktail was added to cells for 30 minutes on ice. Then, 4% paraformaldehyde, 0.5% BSA, and 0.1% Triton was applied as described before performing FACS analysis. Graphs above show the percentage of CD86/80, CD40 and MHC II expression on DCs. results shown are means ± sem for three independent experiments *p < 0.05.
