# Supplementary figures and images for: Resolvin E1 Regulates Th17 Function and T Cell Activation
Source: Front Immunol. 2021 Mar 17;12:637983. doi: 10.3389/fimmu.2021.637983 (PMC8009993; doi:10.3389/fimmu.2021.637983)

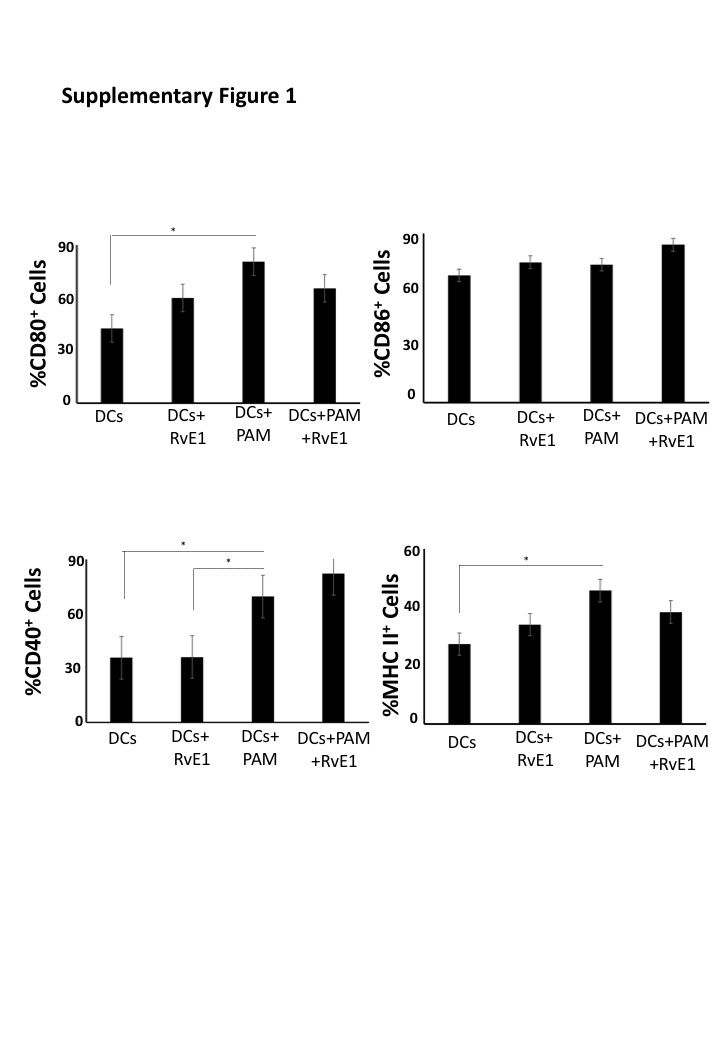

Supplement: Supplementary Figure 1 — CD11c+ dendritic cells (2x10^5 cells/500 𝓂l) were treated with 10 nM RvE1 for 24 hours and then induced with 100 ng PAM3CSK4 for 48 hours. One more dose of RvE1 was applied on day 2. Cells were collected for analysis on day 3; antibody cocktail was added to cells for 30 minutes on ice. Then, 4% paraformaldehyde, 0.5% BSA, and 0.1% Triton was applied as described before performing FACS analysis. Graphs above show the percentage of CD86/80, CD40 and MHC II expression on DCs. results shown are means ± sem for three independent experiments *p < 0.05. [file Image_1.jpeg]
